# The Effect of Using Different Competence Frameworks to Audit the Content of a Masters Program in Public Health

**DOI:** 10.3389/fpubh.2015.00143

**Published:** 2015-05-19

**Authors:** Roger A. Harrison, Isla Gemmell, Katie Reed

**Affiliations:** ^1^Centre for Epidemiology in the Institute for Public Health, The University of Manchester, Manchester, UK

**Keywords:** public health education, public health competences, professional competences, masters in public health, accreditation

## Abstract

**Objectives:**

(1) To quantify the effect of using different public health competence frameworks to audit the curriculum of an online distance learning MPH program, and (2) to measure variation in the outcomes of the audit depending on which competence framework is used.

**Study Design:**

Retrospective audit.

**Methods:**

We compared the teaching content of an online distance learning MPH program against each competence listed in different public health competence frameworks relevant to an MPH. We then compared the number of competences covered in each module in the program’s teaching curriculum and in the program overall, for each of the competence frameworks used in this audit.

**Results:**

A comprehensive search of the literature identified two competence frameworks specific to MPH programs and two for public health professional/specialty training. The number of individual competences in each framework were 32 for the taught aspects of the UK Faculty of Public Health Specialist Training Program, 117 for the American Association of Public Health, 282 for the exam curriculum of the UK Faculty of Public Health Part A exam, and 393 for the European Core Competencies for MPH Education. This gave a total of 824 competences included in the audit. Overall, the online MPH program covered 88–96% of the competences depending on the specific framework used. This fell when the audit focused on just the three mandatory modules in the program, and the variation between the different competence frameworks was much larger.

**Conclusion:**

Using different competence frameworks to audit the curriculum of an MPH program can give different indications of its quality, especially as it fails to capture teaching considered to be relevant, yet not included in an existing competence framework. The strengths and weaknesses of using competence frameworks to audit the content of an MPH program have largely been ignored. These debates are vital given that external organizations responsible for accreditation specify a particular competence framework to be used. Our study found that each of four different competence frameworks suggested different levels of quality in our teaching program, at least in terms of the competences included in the curriculum. Relying on just one established framework missed some aspects of the curriculum included in other frameworks used in this study. Conversely, each framework included items not covered by the others. Thus, levels of agreement with the content of our MPH and established areas of competence were, in part, dependent on the competence framework used to compare its’ content. While not entirely a surprising finding, this study makes an important point and makes explicit the challenges of selecting an appropriate competence framework to inform MPH programs, and especially one which recruits students from around the world.

## Introduction

The Institute of Medicine and the American Public Health Association (1) recommended the use of competence frameworks to inform the content of public health teaching programs. This reflected concerns about variation in the content of public health professional training programs (2), and a growing trend to competence based education in other health professions (3–5). This has resulted in an increase in the number and scope of competence frameworks for public health training and professional practice (1, 6), along with different interpretations of such terms (7). In this paper, we have defined competence to mean a “complex set of measurable behaviors made up of knowledge, skills, and attitudes that can be shown to predict and measure effective performance” (8).

One of the ways in which different competence frameworks for MPH programs are being used is as part of internal and external accreditation processes. Indeed, there is no standardized way within (6, 9), or between countries for assuring the quality of MPH programs. This becomes more challenging as online distance learning has opened the way for students to be registered with a program from another country. Integrating students around the world to an MPH program has the potential to further help prepare graduates to then effectively work across a challenging and diverse global context. It does raise questions about the teaching curriculum, and how to maximize opportunities from a more global student-economy. Part of this needs to be informed by an appropriate quality assurance strategy, which is responsive to a global educational environment, and in meeting the needs of students regardless of where they live and study.

The current study stems from work at The University of Manchester, which has run an online distance learning masters program for 12 years. Each year, this program typically recruits around 70 students from the UK and other European countries, and around 30–40 students from countries outside of the European Union (10). To graduate with an MPH, our students need to pass *three mandatory modules* (Evidence Based Practice, Fundamentals of Epidemiology, and Introduction to Biostatistics) and a *further five* modules, which they select from a choice of 17 optional modules (available at the time of study). Each module reflects 10 weeks of teaching, around 150 study hours, and completion of two, written assignments. This is followed by a 12,000–15,000 word dissertation (11, 12), often relating to students’ vocational experience and future career direction (12). Previous research based on the Manchester MPH found that a diverse virtual student classroom enhanced students’ total learning experience (11). Yet from an educator’s perspective, it creates challenges in ensuring a curriculum which is suitable for all of the MPH students, regardless of where they live, and how this then ought to be quality assured, and by whom. These important questions have not been considered in the public health literature and warrant further investigation.

### Aims

The aim of the current study was to inform the further development and delivery of a high quality online distance learning MPH program. Its primary objective was to complete an audit of the existing curriculum, across the 20 teaching modules, using an appropriate competence framework. Its secondary objective was to quantify the effect on the perceived content of the curriculum when compared using a different competence framework. In particular, we wanted to determine how these varied depending on the optional modules taken by the students.

## Materials and Methods

We reviewed the literature to identify suitable competence frameworks for professional public health education. We wanted to review our course against competence frameworks that reflected countries from which the majority of our students were recruited. The online MPH at University of Manchester includes a broad curriculum covering core aspects relevant to public health professionals. As such, we excluded competence frameworks that focused *entirely* on a specific sub-set of competences [e.g., epidemiology (13) or informatics (14)].

Each item of competence listed in the frameworks was copied into an Excel spreadsheet. This was sent to all module instructors who were given information about how to review the content of their modules against the competences listed in the spreadsheet. A simple coding frame was designed to capture information about the coverage of teaching material in each course module relating to the competences: (1) fully covered, (2) partly covered, (3) comparably covered, and (4) not covered at all. The code “comparably covered” could be used when the direct competence was not covered, but for which comparable teaching material was included. The focus of the primary audit was to identify competences that were not covered at all in any of the teaching material. Therefore, we regrouped the original four categories into two dichotomous variables: (1) competence covered in some way, or (2) competence not covered at all. The module instructors used their expertise to reach a judgment as to which of these four categories was most applicable to each of the competence items presented. The methods for data collection were piloted including the face validity of the competence frameworks in their presented format. Inter-rater reliability was explored with the principal investigator cross checking the results from one of the core modules.

It is difficult to identify any typical groups of students across the program: it is affected by whether they are full or part time, and which optional modules they select, among other things. Consequently, we carried out an analysis using two dummy or model student’s that we created ourselves based on two distinctive possible pathways through the programme. Thus, one student pathway consisted of more quantitative and/or epidemiology focused optional modules. The second pathway focused on more narrative and health promotion focused optional modules. Therefore, our two model students for exploratory purposes with this audit were assumed to consist of:

### Student one

The three mandatory modules (Evidence Based Practice, Fundamentals of Epidemiology & Introduction to Biostatistics) plus five optional modules, which were Communicable Disease Control, Advanced Epidemiology, Evidence Based Public Health, Impact Information Evaluation, and Health Economics.

### Student two

The three mandatory modules (Evidence Based Practice, Fundamentals of Epidemiology & Introduction to Biostatistics) plus five optional modules, which were Health Promotion Theory Methods, Management, Primary Healthcare, Qualitative Research Methods, and Health Promotion and Prevention.

### Analysis

The analysis examined the proportion of competences deemed to be covered in the teaching content of each module, for each of the competence frameworks. This then allowed the calculation of the number of competence items covered in the three mandatory modules (Evidence Based Practice, Fundamentals of Epidemiology & Introduction to Biostatistics) and for the total number of competences covered across all of the twenty units available on the program. A sub analysis then focused on the outcome when applied to a potential learning experience using two model students.

## Results

### Competence frameworks

The literature review identified two competence frameworks specific to MPH education. The Association of Public Health in America competence framework (ASPH) consisted of 117 competence items (15), and the European Core Competencies for MPH Education (ECCMPHE) had 393 competences listed (8). We also included two frameworks adapted for the audit, based on the UK Faculty of Public Health Part A membership exam (FPH) with 282 competences (16), and the educational/knowledge domains from the UK Specialist Registrars Training in Public Health (STP) with 32 listed items (16) (Table 1). Thus in total, the audit covered 824 competence items that were assessed against the curriculum for each of the 20 course modules available on the entire MPH program.

**Table 1:** **The number of competence items covered in each of the competence frameworks**.

Framework	No. of items
Association of Public Health (ASPH) (15)	117
European Core Competencies for MPH Education (ECCMPHE) (8)	393
Faculty of Public Health Part A Exam, UK (FPH) (17)	282
Specialist Training Program, UK (STP) (18)	32
Total across all four frameworks	824

### Audit of three mandatory modules

The first stage of the analysis was restricted to the three mandatory modules in the online MPH program. The proportion of competences that were identified as been covered (fully, partly or comparably) in the teaching material for each of the four competence frameworks were 50% (*n* = 16) for the STP, 30% (118) for the ECCMPHE, 29% (*n* = 34) for ASPH, and 23% (*n* = 66) for the FPH (Table 2).

**Table 2:** **The number (%) of competences included in the curriculum for the three *mandatory* course modules (Evidence Based Practice, Introduction to Biostatistics, and Fundamentals of Epidemiology)**.

Framework	Competences covered in some way in the curriculum^a^ for the three MANDATORY course modules (%)^b^	Competences not included at all in the curriculum^a^ of any of the three MANDATORY course modules (%)^b^
ASPH	34 (29)	83 (71)
ECCMPHE	118 (30)	275 (70)
FPH	66 (23)	216 (77)
STP	16 (50)	16 (50)

*^a^The analysis combined the categories of (a) partly covered, (b) comparably covered, or (c) fully covered)*.

*^b^The percentages are the number of competences addressed in some way across all of the 17 course modules, as a proportion of the total number of competences in each competence framework*.

### Audit of the three mandatory modules plus all of the possible 17 optional modules

The second stage of the analysis focused on the content *across the* entire teaching program (i.e., the three mandatory modules and all of the 17 optional modules (although in practice, a student would take the three mandatory and five optional modules). In this analysis, the proportion of competences that were identified as been covered (fully, partly or comparably) in the teaching material for each of the four competence frameworks were 96% (*n* = 377) for the ECCMPHE, 95% (*n* = 267) for the FPH, 91% (*n* = 29) for the STP, and 88% (*n* = 103) for the ASPH (Table 3).

**Table 3:** **The number (%) of competences included in the curriculum across all 20 course modules available**.

Framework	Competences covered in some way in the curriculum^a^ across the 20 course modules as a whole (%)^b^	Competences not included at all in the curriculum of any of the 20 course modules (%)^b^
ASPH	103 (88)	14 (12)
ECCMPHE	377 (96)	16 (4)
FPH	267 (95)	15 (5)
STP	29 (91)	3 (9)

*^a^The analysis combined the categories of (a) partly covered, (b) comparably covered, or (c) fully covered*.

*^b^The percentages are the number of competences addressed in some way across all of the 20 course modules, as a proportion of the total number of competences in each competence framework*.

### Audit of two example student pathways

The final analysis used two model/possible student pathways through the program to explore the effect of using the different competence frameworks on the outcomes from the audit (Table 4).

**Table 4:** **The number (%) of competences included in the curriculum for two proxy student pathways through the course**.

Framework	Proxy Student Pathway	Competences covered in the curriculum in some way^a^ (%)^b^	Competences not included at all in the curriculum for the two different student pathways (%)^b^
ASPH	Student One	57 (48)	60 (52)
	Student Two	90 (77)	27 (23)
ECCMPHE	Student One	252 (64)	141 (36)
	Student Two	254 (65)	139 (25)
FPH	Student One	128 (45)	154 (55)
	Student Two	186 (66)	96 (34)
STP	Student One	23 (72)	9 (28)
	Student Two	27 (84)	5 (16)

*^a^The analysis combined the categories of (a) partly covered, (b) comparably covered, or (c) fully covered*.

*^b^The percentages are the number of competences addressed in some way across the three mandatory course modules and five optional modules, chosen by two different student pathways, as a proportion of the number of competences in each different framework*.

For Student One (the quantitative/epidemiology focused pathway), the curricula covered 72% (*n* = 23) of competences for the STP framework, 64% (*n* = 252) for the ECCMPHE, 48% (*n* = 57) for the ASPH, and 45% (*n* = 128) for the FPH. For Student Two, this ranged from 84% (*n* = 27) for the STP, 77% (*n* = 90) for the ASPH, 66% (*n* = 186) for the FPH, and 65% (*n* = 254) for the ECCMPHE (Table 4).

## Discussion

All MPH programs need to be fit for purpose, and have the potential to educate students to become effective public health professionals once they graduate. As such, the audit described in the current study is just one of a number of quality assurance initiatives we implement across the teaching program. We found that a detailed audit of each of the 20 modules on an online distance learning MPH was a time consuming process. Each course unit had to assess the content of their module against 824 individual competences. Despite such a task, many of our course leaders reported that it helped them reflect on the content of their program, and to then see links in teaching areas across the different modules. It then led to important discussions about what if any changes needed to be made to the content of the course, including consideration of developing further modules.

One of the main issues when we are to repeat this audit is about deciding on the appropriateness of using just one competence framework. This is the ideal scenario in an effort to reduce time to complete the audit, and we would suggest that this is the most likely option taken by other educationalists who might be/have embarking on such an audit. But the findings of our exploratory audit highlight some of the difficulties in selecting the most ideal framework to use for this purpose. Admittedly, the audit only included four different frameworks, yet this had 824 individual competences to assess the curriculum against, though fewer if it had been restricted to the two frameworks specifically developed for MPH programs. Despite this, we still found important variations in the perceived quality of our program depending on which of the four frameworks was used. We did not find any competence frameworks that were specific to public health in low- and middle-income countries. Of course, MPH programs include many generic skills that can be applied in a wide range of contexts. At the same time, low- and middle-income countries experience particular public health challenges, which require specific approaches for investigation and effective intervention. Their populations remain in desperate need of suitably trained health professionals (19, 20). Such a disconcerting observation has been reported in the recent past (21), and it is encouraging at least to see some further investigation into this topic (22, 23). We also need to ensure that we use competence frameworks relevant to other developing countries beyond north America and the European Union. This will include countries like China, which have dramatically increased the number of students coming to the UK (or studying online) to study for a degree (24).

Clearly, our audit highlighted potential gaps in the content of our MPH program, regardless of which competence framework it was compared against. This was not a surprising finding because the program was never designed to meet all of the competences outlined in any single framework. However, the audit did identify relatively small additions and changes that course leaders made, and provided justification for developing two new modules on the program. At the same time, the audit could not capture all of the teaching included in the program but which is not a competence on any of the frameworks used in this study. These additional areas of teaching are one of the great strengths of this online MPH, providing an extensive learning experience for a wider set of circumstances, and one which we believe is closely aligned with students previous experience, and future aspirations. These strengths are one of the reasons the program was been consistently rated as exemplary by senior external reviewers, along with evidence of extremely satisfied students.

We remain committed to proving an enhanced learning experience for our MPH programs, and that to do otherwise would be a disservice to students and to the populations they then serve. In recent times, we have observed growing pressures for the external accreditation of public health education and even public health departments. This is a complex and challenging debate, although aims such as increasing public accountability while delivering cost-effective interventions are ones we agree are laudable. One concern that is reflected in our current study relates to the methods used to assess an MPH as part of an accreditation process. Thus, we found that in terms of competences, the content of a teaching curriculum could vary in potentially important ways, depending on the competence framework used for its comparison. We stress the importance of this finding, especially when some organizations stipulate a particular competence framework to be used (e.g., Council on Education for Public Health, http://ceph.org/ Accesssed 5 December, 2014). Thus, a curriculum that aims to meet the content of a specific competence framework might be missing out other relevance competences included in a different framework.

A weakness of the current study is that we cannot rule out the possibility of measurement error, with course leaders interpreting the competences in different ways, and with the added challenges of determining what teaching something might actually mean. We accept that we used a relatively basic coding structure and that we might have under or over estimated the content compared with each of the competence frameworks. However, we anticipate that these biases would remain in the same direction across each of the four competence frameworks used in our audit. It is certainly true that our audit helped to stimulate debate across the academic team, and identify potential gaps in the programs content. While there are debates about the value of competence audits/mapping in higher education (25, 26), overall we found this was a positive, albeit time consuming process and it is doubtful that we would use all four frameworks again in future. We also experienced some practical difficulties in that the frameworks were presented in different ways, with lack of clarity if something was an actual competence or a subheading/heading, and with some confusion about the terminology. All of this can easily be rectified, and we encourage any competence frameworks to be presented in a standard format (27, 28) and downloaded as an editable spreadsheet reflecting that used for the Part A exam curriculum of the UK Faculty of Public Health (17). We had a positive response after raising our concerns with a leading author of the ECCMPHE (Birt C, 2014, *personal communication*).

## Conclusion

Few specific competence frameworks exist that can be used to audit the teaching curriculum of an MPH. While time consuming, we found that this process was a positive experience for individual course leaders and the academic team. Our study highlights the challenges and effects of selecting any single competence framework against which the curriculum is assessed. This is greater when recruiting students from other countries, and especially when designing on online distance learning program with students studying concurrently from all over the world. It is important that students, program directors, and organizations responsible for external quality assurance and accreditation are aware of the strengths and weaknesses of these and other approaches for quality assurance. We remain concerned about the apparent lack of specific competence frameworks relevant to MPH education for low- and middle-income countries and others outside of North America and the European Union. At the same time, we would be skeptical of one international competence framework to guide the teaching curriculum of MPH programs around the world, and welcome some level of heterogeneity and flexibility at any one time. We will endeavor to proceed with caution, especially while the evidence to support this approach is wanting.

## Conflict of Interest Statement

The authors declare that the research was conducted in the absence of any commercial or financial relationships that could be construed as a potential conflict of interest.
